# *Orthodenticle* is required for the development of olfactory projection neurons and local interneurons in *Drosophila*

**DOI:** 10.1242/bio.20148524

**Published:** 2014-07-04

**Authors:** Sonia Sen, Silvia Biagini, Heinrich Reichert, K. VijayRaghavan

**Affiliations:** 1 National Centre for Biological Sciences – Tata Institute of Fundamental Research, UAS-GKVK Campus, Bellary Road, Bangalore 560065, India; 2 Biozentrum, University of Basel, Klingelbergstrasse 50/70, CH-4056 Basel, Switzerland; * Present address: FIRC Institute of Molecular Oncology, Via Adamello, 16-20139 Milan, Italy.

**Keywords:** Otd, Olfactory interneuron, Neuroblast, *Drosophila*

## Abstract

The accurate wiring of nervous systems involves precise control over cellular processes like cell division, cell fate specification, and targeting of neurons. The nervous system of *Drosophila melanogaster* is an excellent model to understand these processes. *Drosophila* neurons are generated by stem cell like precursors called neuroblasts that are formed and specified in a highly stereotypical manner along the neuroectoderm. This stereotypy has been attributed, in part, to the expression and function of transcription factors that act as intrinsic cell fate determinants in the neuroblasts and their progeny during embryogenesis. Here we focus on the lateral neuroblast lineage, ALl1, of the antennal lobe and show that the transcription factor-encoding cephalic gap gene *orthodenticle* is required in this lineage during postembryonic brain development. We use immunolabelling to demonstrate that Otd is expressed in the neuroblast of this lineage during postembryonic larval stages. Subsequently, we use MARCM clonal mutational methods to show that the majority of the postembryonic neuronal progeny in the ALl1 lineage undergoes apoptosis in the absence of *orthodenticle*. Moreover, we demonstrate that the neurons that survive in the *orthodenticle* loss-of-function condition display severe targeting defects in both the proximal (dendritic) and distal (axonal) neurites. These findings indicate that the cephalic gap gene *orthodenticle* acts as an important intrinsic determinant in the ALl1 neuroblast lineage and, hence, could be a member of a putative combinatorial code involved in specifying the fate and identity of cells in this lineage.

## INTRODUCTION

The accurate wiring of nervous systems is a multifold task that includes precise control over cellular processes such as cell division and cell fate specification, pathfinding and synaptic partner matching to generate appropriate numbers of neurons and glia that target appropriate regions in the brain and make appropriate synaptic contacts within these regions. What are the molecular mechanisms by which developing nervous systems achieve this? The brain of the holometabolous insect *Drosophila melanogaster* is an excellent model to understand these processes.

*Drosophila* neurons are generated by stem cell like precursors called ‘neuroblasts’ (NBs), most of which divide in an asymmetric manner to self-renew and generate a ‘ganglion mother cell’ (GMC), which has the ability to divide once more to give rise to two post-mitotic neural cells (Doe, 1992; Hartenstein et al., 2008). *Drosophila* neurogenesis occurs in two phases. NBs go through a rapid burst of neurogenesis in the embryo to create the much simpler larval brain (Hartenstein and Campos-Ortega, 1984), and after a period of quiescence, they reinitiate neurogenesis in a second, longer postembryonic phase to create the far more complex adult brain (Ito and Hotta, 1992; Pereanu and Hartenstein, 2006; White and Kankel, 1978). During both phases, the lineage-related neurons born from a single NB often fasciculate their outgrowing axons and hence tend to project to and innervate the same target fields in the brain neuropile (Lovick et al., 2013; Pereanu and Hartenstein, 2006; Wong et al., 2013). Due to these developmental processes, the mature fly brain is a strikingly modular structure with NB lineages representing the ‘modules’ that underlie the basic architecture of the brain's macrocircuitry.

During embryonic development, identifiable NBs form in a highly stereotypical manner at defined locations within the neuroectoderm (Doe, 1992; Hartenstein and Campos-Ortega, 1984; Hartenstein et al., 1987). This stereotypy in the formation and specification of embryonic NBs has been attributed, in part, to the action of embryonic patterning genes that initially define the body axes but are also expressed later in development in unique combinations in the NBs of the embryo (Skeath and Thor, 2003; Urbach and Technau, 2004). Loss-of-function studies performed for some of these genes have revealed that they have important roles in the development of the embryonic brain (Kuert and Reichert, 2013; Lichtneckert and Reichert, 2008; Urbach and Technau, 2008). Examples for this are the two cephalic gap genes *orthodenticle* (*otd*) and *empty spiracles* (*ems*). Like other cephalic gap genes, *otd* and *ems* are first expressed in broad stripes in the anterior region of the embryo at the early blastoderm stage (Dalton et al., 1989; Finkelstein et al., 1990; Walldorf and Gehring, 1992). In their absence, entire embryonic head segments fail to be specified resulting in ‘gaps’ in the head of the embryo (Cohen and Jürgens, 1990; Schmidt-Ott et al., 1994). Subsequently, during embryonic neurogenesis, these two homeodomain transcription factors are expressed in specific sets of embryonic NBs in the central brain and are required for the appropriate development of the embryonic brain regions that derive from these NBs (Hartmann et al., 2000; Hirth et al., 1995; Urbach and Technau, 2003; Younossi-Hartenstein et al., 1997).

Recent work indicates that some of these early “embryonic” patterning genes are also required in specific NB lineages during postembryonic brain development. For example, the cephalic gap gene *ems* has been shown to act in two of the NB lineages that generate the adult-specific olfactory interneurons of the antennal lobe. The antennal lobe, the primary centre for olfactory processing, is made up of dense synaptic regions called glomeruli comprising uniglomerular and multiglomerular projection neurons (PNs) that project to the protocerebrum as well as oligoglomerular and multiglomerular local interneurons (LNs) that do not leave the antennal lobe (Chou et al., 2010; Das et al., 2008; Jefferis et al., 2001; Lai et al., 2008). Most of these interneurons are generated postembryonically by five identified NBs. These are the anterodorsal NB [ALad1 (Jefferis et al., 2001)], the lateral NB [ALl1 (Chou et al., 2010; Das et al., 2008; Lai et al., 2008)], the ventral NB [ALv1 (Lai et al., 2008)], the ventral-LN NB [ALv2 (Das et al., 2011; Lai et al., 2008)] and the ALlv1 (Das et al., 2013; Pereanu and Hartenstein, 2006). The cephalic gap gene *ems* is expressed in two of these NBs in the larval brain. In the ALad1 NB lineage, *ems* is required for correct dendritic targeting of uniglomerular PNs in the antennal lobe (Lichtneckert et al., 2008). In the ALl1 NB lineage, *ems* is required for NB survival; in the absence of *ems* the NB undergoes apoptosis, and therefore no progeny is generated (Das et al., 2008).

Here we focus on the ALl1 NB lineage and show that a second cephalic gap gene, *otd*, is also required in this antennal lobe lineage during postembryonic brain development. We use immunolabelling to demonstrate that Otd is expressed in the NB of this lineage during postembryonic larval stages. Subsequently, we use MARCM clonal mutational methods to show that a large majority of the postembryonic neuronal progeny in the ALl1 lineage undergoes apoptosis in the absence of *otd*. Moreover, we demonstrate that the neurons that survive in the otd loss-of-function condition display severe targeting defects in both the proximal (dendritic) and distal (axonal) neurites. The identification of *otd* as a second cephalic gap gene that is involved in the specification of the ALl1 lineage implies that Otd together with Ems act as important intrinsic determinants and, hence, could be members of a putative combinatorial code involved in specifying the fate and identity of interneurons in the ALl1 lineage.

## MATERIALS AND METHODS

### Fly strains and MARCM analysis

Unless otherwise stated, all flies were obtained from the Bloomington Stock Centre, Indiana, USA. To generate *Tubulin-Gal4* or *GH146-Gal4*, WT or *otd* null clones, females of the following genotypes:

FRT19A/FM7c

y^1^ sn^3^ oc^2^ FRT19A/FM7c

oc^otd-yh13^, FRT19A/FM7c

were crossed with males of the following genotypes:

FRT19A,hsFLP,Tubulin-Gal80; Tubulin-Gal4, UAS-mCD8::GFP/CyO

*FRT19A,hsFLP,Tubulin-Gal80; GH146-Gal4, UAS-mCD8::GFP/CyO*.

The embryos collected from these crosses were aged appropriately at 25°C and were then treated to a 1 hour heat shock regime at 37°C. Heat shocks were given at embryonic (0–16 hours after egg-laying) or early postembryonic (0–4 hours after larval hatching) stages. For the p35 rescue experiments, females of the genotype *oc^otd-yh13^*,*FRT19A/FM7c*; *UAS-p35/CyO* were crossed with males of the genotype *FRT19A*,*hsFLP*,*Tubulin-Gal80*; *Tubulin-Gal4*,*UAS-mCD8::GFP/CyO*. *ems7.1-Gal4* was generated in HR lab.

### Immunohistochemistry

Brains were dissected and stained as described earlier (Wu and Luo, 2006). The primary antibodies used were: rabbit anti-GFP (1:10,000; Molecular Probes, Invitrogen, Delhi, India), chick anti-GFP (1:10,000; AbCam, Cambridge, UK), mouse anti-Bruchpilot (mAbnc82, 1:20; DSHB, Iowa, USA), rabbit anti-Otd (1∶1500, gift from H. Sun University of Taiwan, Taiwan), guinea pig anti-Otd (1∶750, gift from T. Cook, University of Cincinnati School of Medicine, USA). Secondary antibodies – Alexa-488, Alexa-568 and Alexa-647 coupled antibodies generated in goat (Molecular Probes) – were used at 1:400 dilutions.

### Microscopy

Fluorescent preparations were imaged on an Olympus Fluoview (FV1000) scanning confocal microscope. Optical sections were taken at 1 µm intervals with a picture size of 512×512 pixels (or 1024×1024 where required) and digitally processed using Image J (http://rsbweb.nih.gov/ij) and Adobe Photoshop CS3 (Adobe Systems, San Jose, CA, USA).

### Lineage nomenclature

The antennal lobe lineages have been named by various groups in the past. Here we list the various names by which each lineage is called. ALad1 (Ito et al., 2013; Yu et al., 2013)/BAmv3 (Das et al., 2013)/adNB (Jefferis et al., 2001). ALl1 (Ito et al., 2013; Yu et al., 2013)/BAlc (Das et al., 2013)/lNB (Jefferis et al., 2001). ALv1 (Ito et al., 2013; Yu et al., 2013)/BAla1(Das et al., 2013)/vNB (Lai et al., 2008). ALv2 (Ito et al., 2013; Yu et al., 2013)/BAla2 (Das et al., 2013)/vlLN (Das et al., 2011). ALlv1 (Ito et al., 2013; Yu et al., 2013)/BAlp4 (Das et al., 2013).

## RESULTS

### Otd is expressed in the ALl1 neuroblast during postembryonic development

**Fig. 1.**
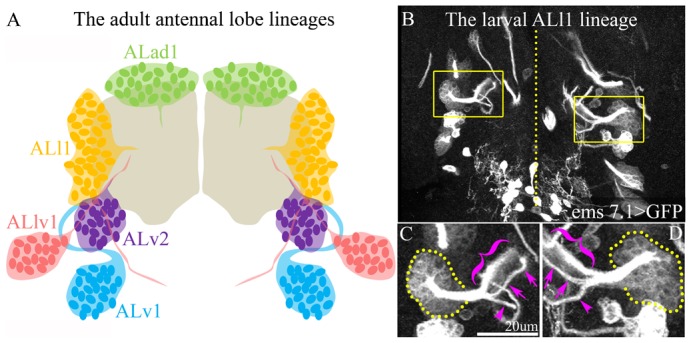
The postembryonic ALl1 lineage is identifiable in the larval brain. (A) The NB lineages that make up the adult antennal lobe are schematized. The ALad1 NB lineage (green) gives rise to typical PNs; ALl1 lineage (orange) gives rise to LNs, typical and atypical PNs; ALv2 lineage (purple) gives rise to glutamatergic LNs; ALv1 lineage (blue) gives rise to atypical PNs and LNs; ALlv1 (pink) gives rise to atypical PNs that also innervate the SOG. These lineages are uniquely identifiable in the larval brain. The ALl1 lineage has a unique secondary axon tract that is labelled by the *ems7.1-Gal4* line. (B) The brain of *ems7.1-Gal4>mCD8GFP* larvae. The yellow boxes highlight the ALl1 lineage on either brain hemispheres separated by the midline (yellow dotted line). These have been magnified in panels C and D and show the stereotypy in their axon tracts. Yellow dotted lines in panels C and D represent the cell bodies of the ALl1 lineage. The magenta parentheses and arrows point to the typical trifurcation of the axon tract of the ALl1 lineage near the larval antennal lobe. Genotype in panels B–D: *ems7.1-Gal4/UASmCD8::GFP*. Grayscale: anti-GFP. Scale bar: 20 µm.

In the mature adult brain, the neuronal cell bodies of the 5 NB lineages that generate the bulk of the antennal lobe interneurons are clustered in 5 groups surrounding each antennal lobe in the deutocerebrum. The ALad1 cell body cluster is located anterodorsal to the antennal lobe, the ALl1 cell body cluster is located lateral to the antennal lobe, and the ALlv1, ALv2 and ALv1 cell body clusters are located ventrolaterally to the antennal lobe (Fig. 1A). The adult-specific “secondary” neurons in these lineages are generated by their parent NB during larval life, and for each NB the lineally related neurons can be identified in the larval brain based on the specific projection pattern of their secondary axon tracts (Lovick et al., 2013; Pereanu and Hartenstein, 2006; Wong et al., 2013).

The adult-specific neurons of the ALl1 lineage project their secondary axons into a unique and highly stereotyped axon tract that initially projects medially in the deutocerebrum and subsequently splits into three branches, two of which project towards the protocerebrum while the third remains in the deutocerebrum (Fig. 1B–D). In addition to its unique secondary axon tract trajectory, this lineage is also labelled by the *ems7.1-Gal4* line, which allows unambiguous identification of the ALl1 lineage in the larval brain (Fig. 1B–D).

**Fig. 2.**
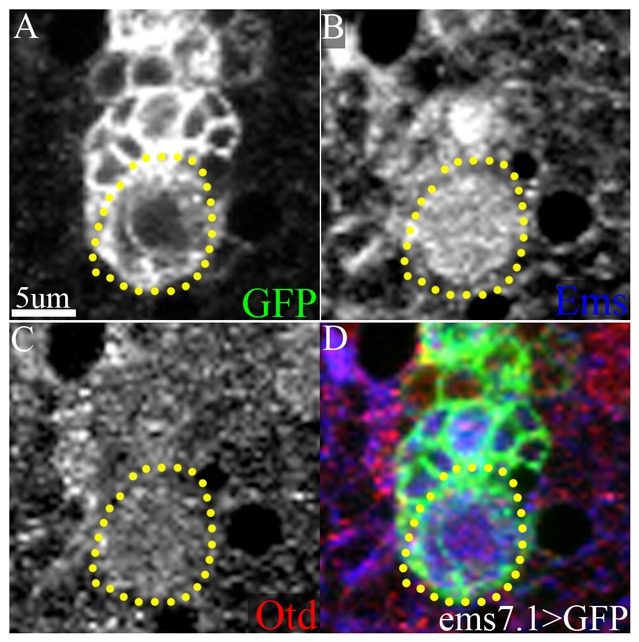
Otd is expressed in the postembryonic ALl1 neuroblast. (A,D) The postembryonic ALl1 lineage (green), labelled by *ems7.1>mCD8GFP*. Its NB is demarcated by the yellow dotted lines. This NB expresses Ems (blue) (B) and co-expresses, albeit at a lower level, Otd (red) (C). Genotype: *ems7.1-Gal4/UASmCD8::GFP*. Green: anti-GFP; Red: anti-Otd; Blue: anti-Ems. Scale bar: 5 µm.

Identification of the ALl1 lineage in the larval brain with the *ems7.1-Gal4* line together with anti-Ems immunolabelling showed that the ALl1 NB expresses Ems during postembryonic development (Fig. 2A,B). This confirmed the findings of earlier work (Lichtneckert et al., 2008). Co-immunolabelling with an anti-Otd antibody showed that the Ems-expressing ALl1 NB also expresses the second cephalic gap gene Otd (Fig. 2A,C,D; yellow dotted lines). Although the ALl1 NB was labelled by the Otd antibody, the level of Otd immunoreactivity was lower than the level of Ems immunoreactivity in this NB (Fig. 2B,C). Similar findings were obtained for earlier larval instar stages (data not shown). We conclude that Otd is expressed in the ALl1 NB during larval development.

### Otd is required for the correct number of neurons in the ALl1 lineage

In order to investigate the developmental role of Otd in the ALl1 lineage, we carried out *otd* loss-of-function experiments on this lineage during larval development. As *otd* null mutants are embryonic lethal, we induced wild-type and *otd* null MARCM neuroblast clones at embryonic and early postembryonic stages and assayed the effect of the *otd* loss of function on the neuronal progeny of the ALl1 NB in the adult brain. In our experiments, we used two null alleles of *otd*, *oc^otdYH13^* and *oc^2^* (also known as *oc^JA101^*). *oc^otdYH13^* is an EMS induced mutation, *oc^2^* is an X-ray induced deletion within the gene; both are known to be amorphs (Finkelstein and Perrimon, 1990; Finkelstein et al., 1990; Wieschaus et al., 1992).

**Fig. 3.**
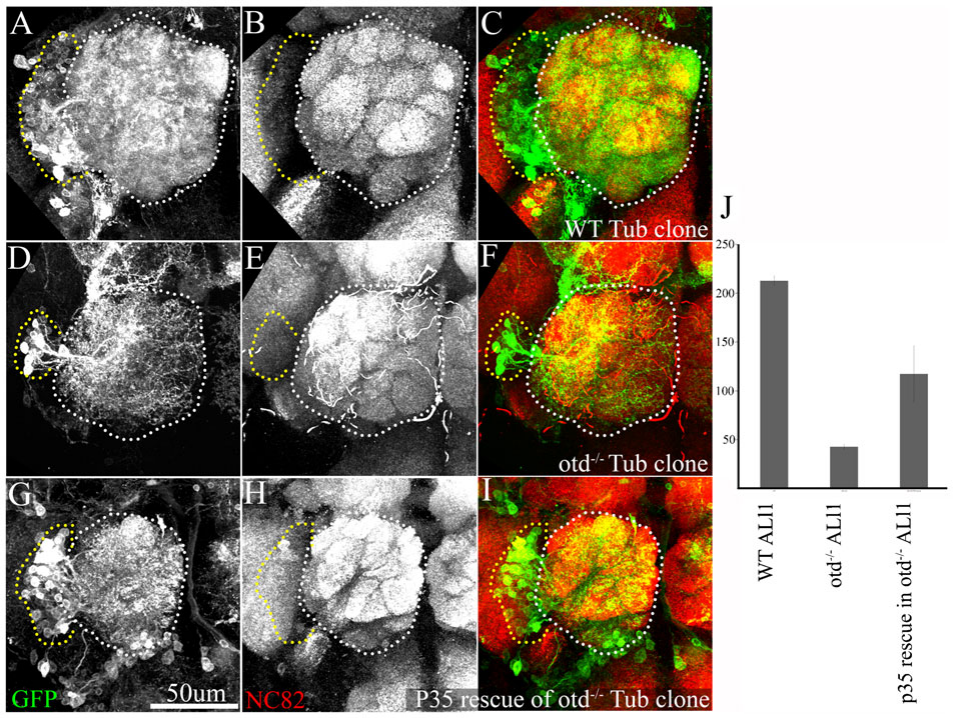
Otd is required for the correct number of neurons in the ALl1 lineage. Tubulin-labelled wild-type MARCM clones (A–C), *otd^−/−^* MARCM clones (D–F) and *p35* rescue in *otd^−/−^* MARCM clones (G–I). The antennal lobes in panels A–I are demarcated by white dotted lines and the cell body cluster of the ALl1 lineage is demarcated by the yellow dotted lines. Note the overall size of the cell body cluster in the wild-type clones of the ALl1 lineage (A–C). This is greatly reduced in *otd^−/−^* clones (D–F) and recovers to some extent in the *p35* rescue in *otd^−/−^* MARCM clones (G–I). (J) A quantification of cell numbers in each of these genotypes. While wild-type clones of the ALl1 lineage have an average of 212.75 cells (SEM 4.8; n = 4), *otd^−/−^* clones of the ALl1 lineage have an average of only 42.7 (SEM 14.95; n = 37). In the *p35* rescue in *otd^−/−^* MARCM clones of the ALl1 lineage the average cell number increases to about 117.3 cells (SEM 28.6; n = 14). (A–C) Genotypes *FRT19A/FRT19A*,*Tubulin-Gal80*,*hsFLP*; *Tubulin-Gal4*,*UAS-mCD8::GFP/+*. (D–F) Genotypes *FRT19A*,*otd^YH13^/FRT19A*,*Tubulin-Gal80*,*hsFLP*; *Tubulin-Gal4*,*UAS-mCD8::GFP/+*. (G–I) Genotypes *FRT19A*,*otd^YH13^/FRT19A*,*Tubulin-Gal80*,*hsFLP*; *Tubulin-Gal4*,*UAS-mCD8::GFP/UAS-p35*. Green: anti-GFP; Red: anti-Bruchpilot. Scale bar: 50 µm.

When randomly induced *tubulin*-labelled wild-type neuroblast clones were generated at either embryonic or early postembryonic (0–4 hours after larval hatching – ALH) stages, the set of adult-specific neurons typical for the ALl1 lineage was recovered in the adult brain. These ALl1 neuroblast clones consisted of about 200 cells, which comprised both LNs and PNs, and, as expected for neuroblast clones containing multiglomerular LNs and PNs, these neurons projected dendritic processes in the antennal lobe with multiglomerular innervation patterns (Fig. 3A–C). In *tubulin*-labelled *otd* null neuroblast clones of the ALl1 lineage, a marked reduction in neural cell number and, correspondingly, a marked decrease in innervation of the antennal lobe were observed (Fig. 3D–F). Thus, whereas wild-type ALl1 neuroblast clones consisted of approximately 200 cells, the *otd* null ALl1 neuroblast clones consisted of only about 40 cells, representing 20 percent of the size of the WT lineage (Fig. 3J). Of the 40 *otd* null ALl1 clones analysed, 11 were embryonically generated and 29 were generated in the early first larval instar. This mutant phenotype was observed in 37/40 *otd* null ALl1 clones suggesting that *otd* is required during postembryonic development. However, this does not rule out the requirement of *otd* during embryonic development as well.

To test if the reduction of cell number in the *otd* mutant ALl1 lineage was due to apoptosis in the lineage, we targeted expression of the anti-apoptotic protein p35, which is a pancaspase inhibitor, in *otd* null ALl1 clones. Clones were generated at the embryonic stage and recovered in the adult brain. When apoptosis was blocked in *tubulin*-labelled *otd* null clones, we observed a partial rescue of the cell loss phenotype in the adult brain (Fig. 2G–I). Of the 76 brains examined, 14 had clones in the ALl1 lineage, and of these, 10 had a marked increase in cell number as compared to the *otd* null ALl1 lineage. Thus the ALl1 clones consisted of about 120 cells, which is three times more than the cell numbers in the *otd* null ALl1 clones (Fig. 2J). The rescue of cell number in the *otd* null ALl1 lineage, following misexpression of an anti-apoptotic gene suggests that the loss of cells in the mutant lineage is due to apoptosis. It is likely that this apoptosis occurs in the neural progeny of this lineage following their generation; however, precocious loss of the neuroblast due to cell death during larval development cannot be completely ruled out.

### Otd is required for correct dendritic targeting of neurons in the ALl1 lineage

The surviving neurons in *tubulin*-labelled *otd* null ALl1 clones manifested abnormal dendritic projection patterns in the antennal lobe. Moreover they also manifested aberrant misprojections outside the antennal lobe towards the suboesophageal ganglion (SOG) and other brain regions. To characterize these misprojection patterns in more detail, we focused our analysis on *GH146-Gal4*-labelled neuroblast ALl1 clones, since *tubulin* drives expression in multiple cell types making documentation of misprojections difficult.

**Fig. 4.**
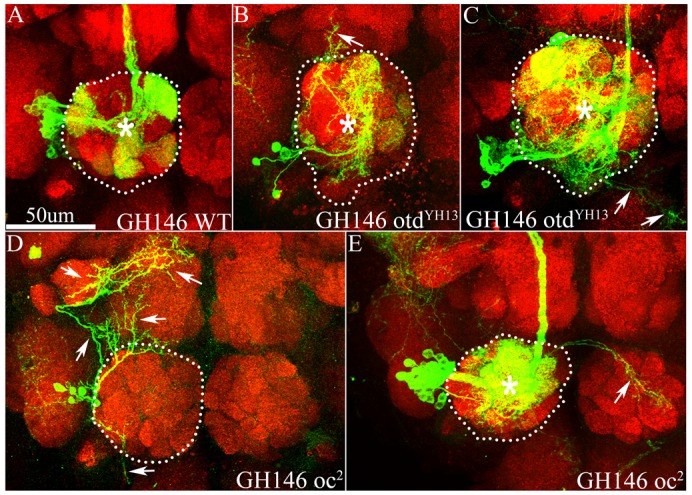
Otd is required for the targeting of the dendrites of ALl1 neurons. (A) A clone of the wild-type ALl1 lineage labelled by *GH146*-*Gal4*, which labels all the typical PNs of the lineage. Note that in the antennal lobe (demarcated by white dotted lines) the PNs restrict their dendrites to the confines of the glomeruli and do not display diffuse, multiglomerular innervations. (B–E) *GH146*-labelled *otd^−/−^* clones of the ALl1 lineage. Note that in these clones the dendrites of the PNs fail to respect glomerular boundaries and have diffuse, multiglomerular innervations in the entire antennal lobe (compare the area around the asterisk in panels B, C and E with the area around the asterisk in panel A). Other mutant phenotypes include innervations of non-antennal neuropiles (arrows, B,C,D) and innervations in the contralateral antennal lobe (arrow, E). (A) Genotypes *FRT1A/FRT19A*,*Tubulin-Gal80*,*hsFLP*; *GH146-Gal4*,*UAS-mCD8::GFP/+*. (B,C) Genotypes *FRT19A*,*oc^otdYH13^/FRT19A*,*Tubulin-Gal80*,*hsFLP*; *GH146-Gal4*,*UAS-mCD8::GFP/+*. (D,E) Genotypes *FRT19A*,*oc^2^/FRT19A*,*Tubulin-Gal80*,*hsFLP*; *GH146-Gal4*,*UAS-mCD8::GFP/+*. Green: anti-GFP; Red: anti-Bruchpilot. Scale bar: 50 µm.

In GH146-labelled neuroblast clones of the wild-type ALl1 lineage, multicellular clones comprising uniglomerular PNs innervating a specific subset of glomeruli were recovered as described previously (Das et al., 2008; Lai et al., 2008) (Fig. 4A). In GH146-labelled *otd* mutant neuroblast clones, the surviving neurons of the ALl1 lineage formed a more diffuse, multiglomerular innervation in the antennal lobe, and often no glomerular boundaries were distinguishable (Fig. 4B,C,E). This mutant dendritic innervation pattern contrasted with the innervation pattern of wild-type *GH146-Gal4*-labelled PNs, which have dendritic innervations within discrete glomeruli (compare Fig. 4A with Fig. 4B,C,E). A variety of other defects were also visible in the surviving mutant PNs. Wild-type GH146-labelled PNs of the ALl1 lineage do not innervate the contralateral antennal lobe; however, in *otd* null clones mutant PNs formed misprojections to the contralateral antennal lobe (Fig. 4E, arrow). PNs in the *otd* null ALl1 lineage also often sent misprojections outside the antennal lobe neuropile to neighbouring neuropiles (Fig. 4B–D, arrows). Occasionally mutant clones had only sparse innervation in the antennal lobes and largely innervated non-antennal neuropile in the protocerebrum (Fig. 4D).

### Otd is required for correct axonal targeting of neurons in the ALl1 lineage

**Fig. 5.**
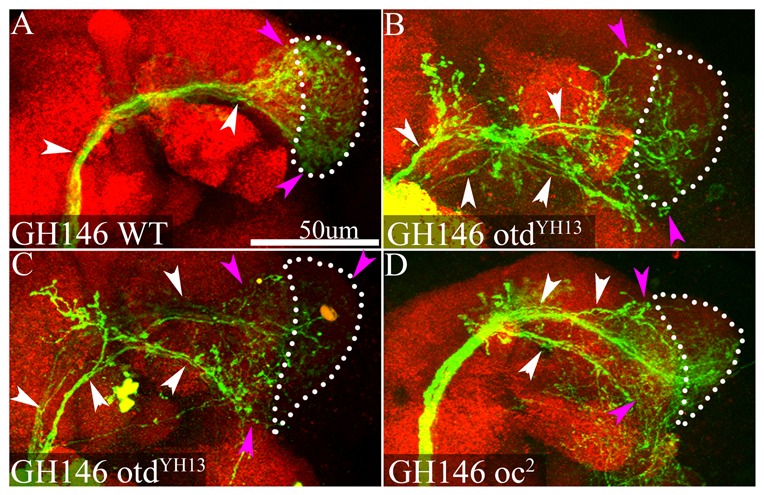
Otd is required for the targeting of the axonal terminals of the ALl1 neurons. (A) The axonal terminals of a wild-type ALl1 lineage labelled by *GH146*-*Gal4* in the protocerebrum. White arrowheads point to the iACT, which is the axonal tract that typical PNs follow to the protocerebrum. Note that this tract remains fasciculated in a single bundle until it approaches the lateral horn in the protocerebrum (white dotted lines). (B–D) The axonal tracts of *GH146*-labelled *otd^−/−^* clones of the ALl1 lineage in the protocerebrum. Note that in these clones the axonal tract of the PNs defasciculate extensively and precociously (white arrowhead, B–D). Other mutant phenotypes include innervations not restricted to the lateral horn (white dotted lines; magenta arrowheads, B–D). (A) Genotypes *FRT1A/FRT19A*,*Tubulin-Gal80*,*hsFLP*; *GH146-Gal4*,*UAS-mCD8::GFP/+*. (B,C) Genotypes *FRT19A*,*oc^otdYH13^/FRT19A*,*Tubulin-Gal80*,*hsFLP*; *GH146-Gal4*,*UAS-mCD8::GFP/+*. (D) Genotypes *FRT19A*,*oc^2^/FRT19A*,*Tubulin-Gal80*,*hsFLP*; *GH146-Gal4*,*UAS-mCD8::GFP/+*. Green: anti-GFP; Red: anti-Bruchpilot. Scale bar: 50 µm.

The surviving neurons in *tubulin*-labelled *otd* null ALl1 clones also manifested abnormal axonal projection patterns in the protocerebrum. To characterize these axonal defects, we again focused on *GH146-Gal4*-labelled clones. In wild-type neuroblast clones, the *GH146*-labelled PNs of the ALl1 lineage projected their axons via the inner antennocerebral tract (iACT) to the protocerebrum and form axonal terminals in the calyx of the mushroom body and in the lateral horn as previously reported (Marin et al., 2002). Throughout most of the protocerebrum, the labelled wild-type PN axons ran as a single fascicle, which only defasciculated in the lateral horn (Fig. 5A).

Surviving *otd* null PNs in the ALl1 lineage resembled wild-type PNs in their gross anatomy; their axonal trajectory resembled the trajectory normally taken by PNs, they appeared to innervate the lateral horn, and on occasion they even had innervations in the calyx of the mushroom body. However, the axonal arborizations of mutant PNs in the protocerebrum manifested marked defects. Fig. 5B–D shows three examples of the axonal arborizations of *otd* null PNs in the lateral protocerebrum. In all three *otd* null clones, the axon bundle defasciculated extensively much before the lateral horn (white arrows). In addition to this, the defasciculated axons manifested extensive innervation of inappropriate neuropile regions not restricted to the lateral horn (magenta arrows). A quantification of the occurrence of all mutant phenotypes in the ALl1 lineage is given in Table 1.

**Table 1.**
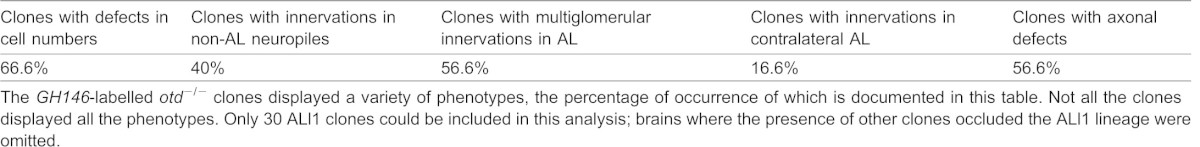
Percentages of *otd*^−/−^ ALl1 clones that display the various mutant phenotypes

Taken together, these data indicate that *otd* is required in the ALl1 NB for the appropriate targeting of both the proximal dendritic arbors and distal axonal terminals of the surviving neurons of this lineage. This, together with the requirement of Otd for the correct number of neurons in the ALl1 lineage implies that the cephalic gap gene *otd*, like the co-expressed cephalic gap gene *ems*, is essential for the development of olfactory interneuron circuitry.

### Otd is not required in the postmitotic neurons for targeting in the ALl1 lineage

**Fig. 6.**
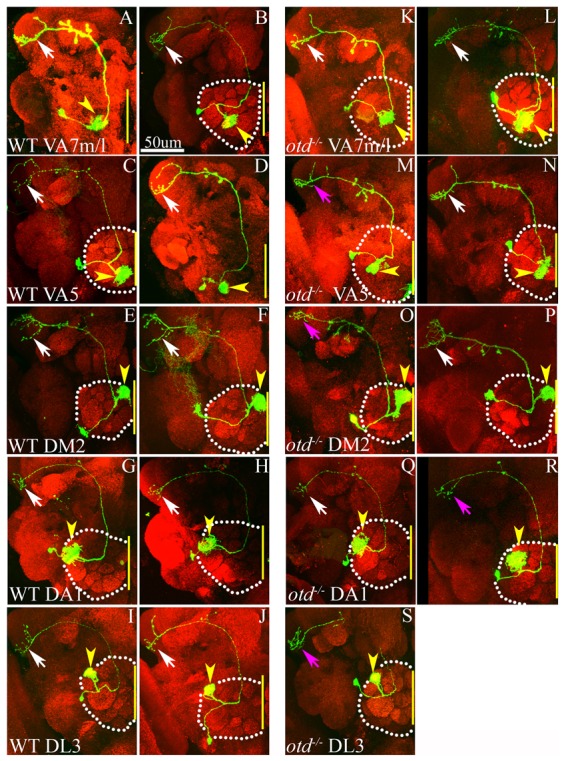
Otd is not required in the postmitotic neurons for the targeting of the ALl1 PNs. (A–J) Wild-type single cell PN clones of the ALl1 lineage labelled by *GH146*-*Gal4*. These clones were generated during the second larval instar stage and five classes of PNs were recovered, namely, VA1l/m (A,B), VA5 (C,D), DM2 (E,F), DA1 (G,H) and DL3 (I,J). These PN are named according to the glomeruli they innervate (yellow arrowheads, A–J). The axons of these PNs project into the protocerebrum via the inner antennocerebral tract and innervate the lateral horn. The innervation pattern of the axonal terminals in the lateral horn for each of these PN classes is highly stereotyped (white arrows, A–J). (K–S) Corresponding *otd* null single cell PN clones of the same PN classes. The dendritic innervations in these glomeruli are comparable to wild type (yellow arrowheads, K–S) as are the axonal innervations in the lateral horn (white arrows, K–S). Occasionally, innervation patterns in the lateral horn deviate from the stereotyped innervations seen in the wild type; however, these are rare (magenta arrowheads, M,O,R,S). (A–J) Genotypes *FRT1A/FRT19A*,*Tubulin-Gal80*,*hsFLP*; *GH146-Gal4*,*UAS-mCD8::GFP/+*. (K–S) Genotypes *FRT19A*,*oc^otdYH13^/FRT19A*,*Tubulin-Gal80*,*hsFLP*; *GH146-Gal4*,*UAS-mCD8::GFP/+*. Green: anti-GFP; Red: anti-Bruchpilot. Yellow lines indicate the midline. Scale bar: 50 µm.

In order to determine if *otd* was required in postmitotic neurons of the ALl1 lineage, we generated *otd* null, *GH146-Gal4*-labelled, single cell clones in the second larval instar stage and analysed the axonal and dendritic projection patterns of the resultant single cell clones in the adult brain. These experiments revealed two significant findings. Firstly, we noticed a marked reduction in clonal frequencies of the wild-type and *otd* null single cell clones. In the wild type we recovered 34 single cell clones in 131 brains examined (clonal frequency ∼26%); in otd mutant clones we recovered only 40 single cell clones out of 310 brains examined (clonal frequency ∼13%). Secondly, in the surviving *otd* null single cell clones of the ALl1 lineage, we did not see any marked targeting defects. Thus, the 34 wild-type single cell clones represented 5 different PN classes – VA7m/l, VA5, DM2, DA1 and DL3 (Fig. 6A–J). As previously reported, these PN classes, which are defined by their specific dendritic innervations in discrete glomeruli of the antennal lobe, also had unique and stereotyped axonal innervations in the lateral horn (Lin et al., 2012). These five PN classes were also represented in the *otd* null single cell clones (Fig. 6K–S). The dendrites of these single cell clones innervated the appropriate glomeruli and the axons projected to the protocerebrum via the inner antennocerebral tract to innervate the lateral horn. In the lateral horn, a large majority of the *otd* null PNs retained the class specific innervation pattern. A few exceptions, where the innervations in the lateral horn deviated slightly from the stereotyped wild-type innervation pattern are shown (Fig. 6M,O,R,S, magenta arrows). Taken together these results suggest that *otd* is required in the neuroblast but not in the postmitotic neurons of the ALl1 lineage for the correct axonal patterning of PNs.

## DISCUSSION

During early embryogenesis, the cephalic gap gene *otd* is expressed in a broad stripe in the anterior most domain of the cephalic region of the embryo where it is known to specify the entire segment, including the anterior brain that derives from this segment. Studies that have analysed the expression of *otd* in the later stages of embryonic brain development have shown that *otd* continues to be expressed in specific neuroblasts. For example, in the protocerebral part of the embryonic brain, *otd* is expressed in about 70% of the neuroblasts (Urbach and Technau, 2003). Interestingly, 15% of the embryonic neuroblasts that express *otd* co-express the cephalic gap gene *ems*.

Here we report that *otd* is also co-expressed with *ems* in a neuroblast lineage during postembryonic brain development. We have focused our analysis on the ALl1 neuroblast, which has been shown to express *ems* during larval development. While our findings indicate that the expression of *otd* is relatively low compared to the level of *ems* expression in the ALl1 neuroblast, our mutant analysis indicates that *otd* is essential for the development of the neurons in this lineage. It will be interesting to see if *otd* might be similarly involved in the development of the other neuroblast lineages in the brain.

Mutant analysis of the function of *otd* in the ALl1 lineage revealed several distinct requirements for this gene. The first, most evident defect observed in clonal loss-of-function experiments was the reduction in cell number of the ALl1 lineage; only 20% of the cells present in the wild-type adult brain were seen in the mutant condition. This phenotype is reminiscent of, but not exactly like, the phenotypes observed in this lineage due to the loss of function of three other genes, *empty spiracles* (*ems*), *homothorax* (*hth*) and *extradenticle* (*exd*) (Das et al., 2008; Lichtneckert et al., 2008; Ando et al., 2011). Upon the loss of function of any of these genes, the entire lineage is eliminated. In contrast, upon the loss of function of *otd*, 20% of the neural cells (∼40 cells) survive and are present in the adult brain. This suggests that the mechanism of action of these genes might be different. In this respect, it is interesting to note that accompanied with the loss of function of *ems*, *hth* or *exd* a severe reduction in the size of the antennal lobe results, whereas following *otd* loss of function, the lobe size and its general glomerular organization remains largely unaffected.

A different requirement for *otd* in the ALl1 lineage determined by our mutational analysis was in the targeting of the dendrites and the arborization of the axons of the 20% of the cells that do survive to adulthood. Upon the loss of function of *otd*, ALl1 PNs displayed a variety of targeting defects including diffuse and disorganised dendritic arbours, innervations in non-antennal neuropiles, as well as extensive, premature defasciculation and misprojections of the axonal terminals. This suggests that patterning of the PNs at both the proximal and the distal terminals might be coupled. Such coupling of PN pattering has been uncovered for other genes as well, including other transcription factors like *acj6*, *drifter*, *hth*, *exd* and *lola* (Ando et al., 2011; Komiyama et al., 2003; Spletter et al., 2007).

It has been postulated that the identity of a NB and its lineage depends upon a certain constellation of transcription factors that acts as a code of identity (Shirasaki and Pfaff, 2002). Expression analysis of NBs in the embryo has revealed that there do exist unique combinations of transcription factors in specific NBs (Urbach and Technau, 2003). Moreover, recent studies, which are largely limited to a few well-described lineages in the brain, are beginning to identify the elements of putative ‘combinatorial codes’ of NB specification (Bello et al., 2007; Das et al., 2008; Kuert et al., 2012; Lichtneckert et al., 2008). Results from this study imply that the two cephalic gap genes *otd* and *ems* are included among the set of intrinsic cell fate determinants for the ALl1 lineage. As most postembryonic lineages have now been identified in both the larval and adult brains, such molecular genetic analyses can now be extended to other brain lineages (Ito et al., 2013; Lovick et al., 2013; Wong et al., 2013; Yu et al., 2013). It is noteworthy that although analyses such as these have uncovered genes that are required in NB lineages for their survival or local targeting, none, so far, have identified genes that can actually switch the identity of one NB lineage into that of other. It will be interesting to see if future studies uncover such important factors that determine the identities of lineages.
